# High-dose chemotherapy and autologous stem cell transplantation in primary lymphomatoid granulomatosis of the central nervous system

**DOI:** 10.1007/s00432-022-04531-y

**Published:** 2022-12-24

**Authors:** Verena Nilius-Eliliwi, Hannes Treiber, Sabine Seidel, Deepak B. Vangala, Roland Schroers

**Affiliations:** 1 Department of Hematology and Oncology, Knappschaftskrankenhaus, Ruhr-University Bochum, In Der Schornau 23-25, 44892 Bochum, Germany; 2 Department of Hematology and Oncology, University of Göttingen, Göttingen, Germany; 3 Department of Neurology, Knappschaftskrankenhaus, Ruhr-University, Bochum, Germany

**Keywords:** Lymphomatoid granulomatosis (LG), Central nervous system (CNS), High-dose chemotherapy (HCT), Autologous stem cell transplantation (ASCT)

## Abstract

Primary lymphomatoid granulomatosis of the CNS (CNS-LG) is a rare lymphoid neoplasia associated Epstein–Barr Virus (EBV) and often accompanied by immunodeficiencies. No treatment standards have been defined yet. However, due to often devastating neurologic sequelae and based on similarities to diffuse large B-cell lymphoma, curative treatment requires intensive therapy protocols resembling protocols applied in CNS lymphoma. Here, the clinical courses and treatments of four primary CNS-LG patients in analogy to aggressive CNS-lymphomas including methotrexate, thiotepa, cytarabine, carmustine, and rituximab are presented. This is the first report on high-dose chemotherapy with CNS-directed drugs and autologous blood stem cell transplantation in primary CNS-LG.

## Letter to the editor

Lymphomatoid granulomatosis (LG) is an uncommon disease that is classified as mature B-cell neoplasm according to 2016 WHO classification of lymphoid neoplasms (Swerdlow et al. 2016). It predominantly befalls men and is characterized by angioproliferative and angiodestructive growth patterns with infiltration of T-lymphocytes (Katzenstein et al. 1979). LG is associated with seropositivity for Epstein–Barr Virus (EBV). As LG is often diagnosed in immunocompromised patients, an insufficient eradication of EBV has been proposed as a pathophysiological mechanism (Sordillo et al. 1982). According to the distribution of EBV-positive atypical B-lymphocytes, LG can be separated in three grades with scattered EBV-positive cells in grade 1 up to extensive infiltration in grade 3 (Song et al. 2015). In grades 1 and 2, monoclonality as detected by immunoglobulin heavy chain polymerase chain reaction (IGH-PCR) PCR is observed less frequently than in grade 3 (Song et al. 2015). LG affects multiple organ systems; however, more than 90% of patients show manifestations in the lung (Song et al. 2015; Melani et al. 2018). In very rare cases, LG can affect the CNS without any systemic manifestations, therefore described as primary CNS-LG (Katzenstein et al. 1979; Song et al. 2015).

To date, standardized therapies have not been established due to the rare occurrence of the disease. Cure of systemic LG grade 1 and 2 with steroids or rituximab only has been reported (Katzenstein et al. 1979; Zaidi et al. 2004). Furthermore, curative treatment with interferon-alpha has been described, reflecting the underlying disease mechanism of defective EBV eradication and supporting immunostimulation being a reasonable treatment approach (Wilson et al. 1996). In accordance, cessation of immunosuppressants has led to complete remissions in single cases (Aiko et al. 2018; Shimada et al. 2007).

In advanced stages of systemic LG, more intensive treatment regimens such as R-EPOCH, R-CHOP, and other cyclophosphamide-containing protocols have been applied (Chavez et al. 2016; Dunleavy et al. 2010). Also, high-dose chemotherapy with autologous stem cell transplantation (HCT-ASCT) has been reported as successful treatment. An EBMT case series showed that HCT-ASCT is effective in relapsed or refractory LG (Siegloch et al. 2013).

Primary CNS-LG is even rarer; accordingly, treatment standards have not been defined in this special situation. Limited access to biopsies and restricted drug access due to the blood–brain barrier are especially challenging in this situation. Due to similarity of CNS-LG to primary CNS lymphomas (PCNSL), a reasonable rationale is to base the treatment on therapeutic advances of PCNSL. For diffuse large B-cell lymphoma (DLBCL) manifesting as CNS-lymphoma, HCT-ASCT has been well established as effective consolidation therapy with a clearly curative potential (Seidel et al. 2022; Illerhaus et al. 2016; Kasenda et al. 2017). Current protocols usually consist of induction with rituximab and methotrexate (R-MTX; rituximab 375 mg/m^2^, MTX 4 g/m^2^) and rituximab, thiotepa, and cytarabine (R-TT/AraC, rituximab 375 mg/m^2^, cytarabine 3000 mg/m^2^, thiotepa 40 mg/m^2^), respectively. Carmustine (BCNU), thiotepa, and rituximab (R-BCNU/TT; BCNU 400 mg/m^2^, thiotepa 10 mg/kg) followed by reinfusion of autologous hematopoietic cells is a feasible, effective, and often administered HCT protocol in this setting (Seidel et al. 2022; Illerhaus et al. 2016; Kasenda et al. 2017).

Since there is no report on primary CNS-LG successfully treated with HCT-ASCT in the literature, we conducted a retrospective cohort study of patients with primary CNS-LG treated with HCT-ASCT between 2014 and 2021 at our institutions. Patients had been treated in analogy to PCNSL with protocols for induction and high-dose chemotherapy as described previously (Illerhaus et al. 2016). Details of patient data are given in Table 1. All patients suffered from biopsy proven primary CNS-LG without evidence of systemic involvement. PET/CT and bone marrow biopsies were without evidence of systemic manifestations of LG in each case. Two of four patients (pat. 1 and 2, Table 1) were still alive and free of disease with a follow-up time of 28 and 36 months after HCT-ASCT, respectively.

**Table 1:** Study population—Characteristics of four patients with primary CNS lymphomatoid granulomatosis

No	Sex	Age	ECOG	CCI	Grade/clonality	EBV	localization	Induction therapy	Status before HCT-ASCT	Status after HCT-ASCT	DFS
1	f	30	0	0	2 polyclonal	IHC pos IgG pos IgM neg	Left Cerebellar peduncle	2 × R-MTX 2 × R-TT/ AraC	CR	CR alive	28 months
2	m	44	0	1	1 polyclonal	PCR pos IgG pos IgM neg	Diffuse frontobasal and basal ganglia	1 × R-MTX 2 × R-TT/ AraC	PR	CR alive	36 months
3	m	64	1	6	3 monoclonal	IHC pos IgG pos IgM neg	Diffuse	3 × R-TT/ AraC	PR	PR dead	0
4	f	58	2	4	3 monoclonal	IHC pos IgG pos IgM neg	Left cerebellum, supratentorial	1 × R-TT/ AraC	PD	PD dead	0

*AraC* cytarabine, *CCI* Charlson comorbidity index, *CR* complete response, *EBV* Epstein-Barr-virus, *ECOG* Eastern collaborative group, *HCT*-*ASCT* high-dose chemotherapy with autologous stem cell transplantation, *IHC* immunohistochemistry, *MTX* methotrexate, *DFS* disease-free survival, PR partial response, *R* rituximab, *TT* thiotepa

The patients presented with a broad range of neurological symptoms reflecting the different manifestations from diffuse supratentorial to unilocular cerebellar infiltration. Two patients had been treated with immunosuppressive agents before CNS-LG diagnosis: tacrolimus in patient 3 after kidney transplantation and azathioprine in patient 4 because of rheumatic disease (granulomatosis with polyangiitis). Two patients (pat. 3 and 4, Table 1) had been diagnosed with grade 3 disease with monoclonality shown by IGH-PCR. Patient 1 had polyclonal grade 2 and patient 2 polyclonal grade 1 disease. In all 4 patients, the biopsy samples showed evidence of EBV, either immunohistochemically or by PCR (Table 1).

Originally, all patients were planned to receive 2 cycles of R-MTX and 2 cycles R-TT/AraC as induction chemotherapy, respectively. However, patients 3 and 4 had contraindications against MTX (renal impairement and history of MTX-induced pneumonitis). Accordingly, patient 3 received 3 cycles of R-TT/AraC. Patient 4 exhibited massive LG progression following the first course of R-TT/AraC. Therefore, she proceeded to HCT-ASCT directly thereafter. Also, patient 2 did not respond well to R-MTX. Overall, sufficient numbers of autologous hematopoietic cells could be collected early after cycle one or two of the induction therapy.

HCT protocols for all patients comprised rituximab, carmustine, and thiotepa in the above stated dosage (Illerhaus et al. 2016). Prior to HCT two patients showed partial response (PR), one complete response (CR), and one progressive disease (PD) on restaging MRIs, respectively. All patients developed at least mild infectious complications post HCT-ASCT. Patient 3 died 50 days after HCT-ASCT due to refractory pneumonia based on severe immunodeficiency. Patient 4 who had entered HCT-ASCT during LG progression died 5 days after transplantation with fulminant disease deterioration. Two patients (1 and 2) were alive and free of LG-disease at study analysis (Table 1). They had an ECOG of 0 before HCT-ASCT and the Charlson comorbidity index (CCI) scorings were 2 and 3, respectively. On the contrary, patients 3 and 4 had ECOGs of 1 and 2, and the CCI scores were 8 and 6, respectively (Table 1).

LG is rare and manifests as a systemic disease, mostly involving the lungs. CNS involvement is infrequent but as in other hematological disorders poses special challenges to treatment protocols, because of limited CNS accessibility for many cytotoxic drugs. Today, therapeutic strategies based on large clinical studies for primary CNS-LG are missing. In analogy to CNS lymphomas, we aimed for treatment of ou CNS-LG patients with protocols containing high-dose MTX, thiotepa, cytarabine, carmustine, and rituximab as previously reported for PCNSL (Illerhaus et al. 2016). The rationale behind this approach is the disease similarity, especially between high-grade LG and DLBCL. Furthermore, eradication of CD20 positive cells by rituximab is reasonable in LG, due to the association with EBV persistence. Though, profound statistics are impossible with a sample size of four patients. Nevertheless, the chosen treatment approach was feasible and effective in patients with primary CNS-LG. Our clinical observations indicate that CNS-penetrating drugs such as MTX, thiotepa, and cytarabine can pass the blood–brain barrier and were effective in inducing remissions in CNS-LG. Besides, HCT-ASCT was an effective consolidative treatment in those patients achieving at least a partial response (pat. 1–3) after induction therapy. By now, only three single case reports of *secondary* CNS-LG who underwent HCT-ASCT have been published (Table 2). Our report represents the first to give treatment and outcome details on a group of *primary* CNS-LG patients.

**Table 2:** Literature review: LG with CNS manifestation and HDT-ASCT

References	Study type	Lymphomatoid granulomatosis (LG) manifestation	Grade/clonality	Prior treatments	Follow-up	HCT regimen	Remission status
Lemieux 2002	Case report	Lungs, secondary CNS	2/3 polyclonal	prednisone, CHOP, ESHAP, 2 × ICE	1 year	BEAM	CR
Siegloch 2013	Single report from a case series	Lungs, liver, adrenal glands, kidney, secondary CNS	1 NA	R-CHOP, MTX, R-IE, AraC	1.5 years	TC/TBI	PR
Fernandez-Alvarez 2014	Case report	Lungs, secondary CNS	3 NA	HD-MTX/Ifo + i.th. MTX	1.5 years	C/TBI	PR

*AraC* cytarabine, *BEAM* carmustine, etoposide, cytarabine, melphalan, C cyclophosphamide, *CHOP* cyclophosphamide, doxorubicin, vincristine, prednisolone, *CR* complete response, *ESHAP* etoposide, methylprednisolone, high dose cytarabine, cisplatin, *ICE* ifosfamide, carboplatin, etoposide, *IE* ifosfamide, etoposide, *Ifo* ifosfamide, *i*.*th*. intrathecal, *MTX* methotrexate, *NA* not available, *R* rituximab, *PR* partial response, *TBI* total body irradiation, *TC* docetaxel, cyclophosphamide

Non-relapse mortality (NRM) following HCT-ASCT remains a major issue, especially in immunocompromised patients (pat. 3). Considering immunodeficiencies in most LG patients, balancing the risk and benefit of intensive treatment schedules including HCT-ASCT is a challenge. In the situation of LG infiltrating the CNS, decision towards intensive treatment protocols can be facilitated considering otherwise devastating neurologic sequelae of uncontrolled CNS-LG. The curative potential of the induction and HCT protocols as proven in PCNSL (Siegloch et al. 2013; Seidel et al. 2022; Illerhaus et al. 2016) and suggested in two of our CNS-LG patients further supports this treatment approach. Unquestionably, the results of our case series require further clinical studies before final recommendation of consolidating HCT-ASCT can be made in eligible CNS-LG patients in first remission.

In summary, this is the first report of successful administration of CNS-directed chemotherapy and HCT-ASCT in a group of primary CNS-LG patients. Treatment in analogy to aggressive CNS-lymphomas including methotrexate, thiotepa, cytarabine, carmustine, and rituximab was feasible and effective. Thorough diagnostic procedures including tumor biopsies and risk assessment regarding immunodeficiency related NRM should be undertaken prior to initiation of intensive CNS-directed treatment including HCT-ASCT.

## Data Availability

All data are made available for sharing.
